# Genetic autonomy and low singlet oxygen yield support kleptoplast functionality in photosynthetic sea slugs

**DOI:** 10.1093/jxb/erab216

**Published:** 2021-05-15

**Authors:** Vesa Havurinne, Maria Handrich, Mikko Antinluoma, Sergey Khorobrykh, Sven B Gould, Esa Tyystjärvi

**Affiliations:** 1 Department of Biotechnology/Molecular Plant Biology, University of Turku, Turku, Finland; 2 Department of Biology, Heinrich-Heine-Universität, Düsseldorf, Germany; 3 University of Exeter, UK

**Keywords:** Kleptoplasty, photoinhibition, photosynthetic sea slugs, PSII repair cycle, reactive oxygen species, singlet oxygen, *Vaucheria litorea*

## Abstract

The kleptoplastic sea slug *Elysia chlorotica* consumes *Vaucheria litorea*, stealing its plastids, which then photosynthesize inside the animal cells for months. We investigated the properties of *V. litorea* plastids to understand how they withstand the rigors of photosynthesis in isolation. Transcription of specific genes in laboratory-isolated *V. litorea* plastids was monitored for 7 days. The involvement of plastid-encoded FtsH, a key plastid maintenance protease, in recovery from photoinhibition in *V. litorea* was estimated in cycloheximide-treated cells. *In vitro* comparison of *V. litorea* and spinach thylakoids was applied to investigate reactive oxygen species formation in *V. litorea*. In comparison to other tested genes, the transcripts of *ftsH* and translation elongation factor EF-Tu (*tufA*) decreased slowly in isolated *V. litorea* plastids. Higher levels of FtsH were also evident in cycloheximide-treated cells during recovery from photoinhibition. Charge recombination in PSII of *V. litorea* was found to be fine-tuned to produce only small quantities of singlet oxygen, and the plastids also contained reactive oxygen species-protective compounds. Our results support the view that the genetic characteristics of the plastids are crucial in creating a photosynthetic sea slug. The plastid’s autonomous repair machinery is likely enhanced by low singlet oxygen production and elevated expression of FtsH.

## Teaser

*Vaucheria litorea* plastids exhibit increases in the relative expression of key plastid maintenance genes upon isolation, and produce little singlet oxygen. These factors likely contribute to plastid longevity in kleptoplastic slugs.

## Introduction

Functional kleptoplasty in photosynthetic sea slugs depends on two major components: the first is a slug capable of stealing plastids and retaining them as functional organelles within its cells, and the second is a plastid with a specific genetic repertoire (de Vries *et al.*, 2015). All kleptoplastic species belong to the clade Sacoglossa (Rumpho *et al.*, 2011; de Vries *et al.*, 2014). These sea slugs are categorized, based on their plastid retention times, as no retention, short-term retention (hours to ~10 days), and long-term retention (≥10 days to several months) species (Händeler *et al.*, 2009). The record-holding species, *Elysia chlorotica*, can retain plastids for roughly a year (Green *et al.*, 2000). The mechanisms utilized by the slugs to selectively sequester plastids from the algae they consume remain uncertain, although recent studies have shown that in *E. chlorotica* it is an active process reminiscent of that observed for symbiotic algae and corals (Chan *et al.*, 2018). The slugs possibly rely on scavenger receptors and thrombospondin type 1 repeat proteins for plastid recognition (Melo Clavijo *et al.*, 2020).

The sacoglossans’ ability to sequester plastids tends to distract attention away from the unique features of the sequestered organelle, which forms the second component of a photosynthetic slug system. Long-term-retention sea slugs are able to maintain functional plastids from only a restricted list of siphonaceous algae, and usually from only one species. Some sacoglossans have a wide range of prey algae, but long-term retention of plastids by these slugs is still limited to specific algal sources (Christa *et al.*, 2013; de Vries *et al.*, 2013). The native robustness of some plastid types was noticed decades ago, and early on was suggested to contribute to their functionality inside animals (Giles and Sarafis, 1972; Trench *et al.*, 1973a, b). Studies focusing on the specific properties of the algal plastids are, however, scarce. Reduction of the plastid genome (the plastome) during evolution has stripped the organelle of many genes required for self-maintenance (Martin, 2003), but genomic analysis of algal plastomes suggests that three genes (*tufA, ftsH*, and *psbA*) could be among those critical for plastid maintenance inside a slug cell (de Vries *et al.*, 2013). Out of the three genes, *psbA* remains present in all plastomes, including those of higher plants, whereas *tufA* and *ftsH* are encoded by most algal plastid genomes (Baldauf and Palmer, 1990; Oudot-Le Secq *et al.*, 2007; de Vries *et al.*, 2013). It has been suggested that the plastid-encoded translation elongation factor EF-Tu (*tufA*) helps maintain translation, specifically of the thylakoid maintenance protease FtsH (*ftsH*) involved in the repair cycle of photosystem II (PSII) (de Vries *et al.*, 2013). FtsH degrades the D1 protein (encoded by *psbA*) of damaged PSII before the insertion of *de novo-*synthesized D1 into PSII (Mulo *et al.*, 2012; Järvi *et al.*, 2015). Without continuous replacement of the D1 protein, light-induced damage to PSII would rapidly curtail photosynthesis (Tyystjärvi and Aro, 1996).

**Fig. 1.**
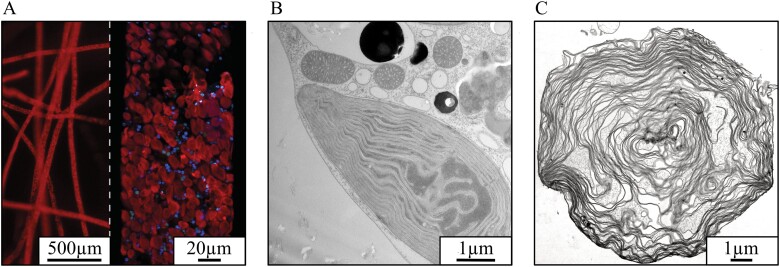
Microscopic images of *V. litorea*, the main source of plastids for the photosynthetic sea slug *E. chlorotica*. (A) Chlorophyll autofluorescence (red) and nucleus-specific dye fluorescence (blue) from *V. litorea* filaments; a detail of a single filament is shown on the right. (B, C) TEM images showing (B) a plastid *in vivo* in a *V. litorea* cell and in close proximity to several mitochondria, and (C) an isolated single plastid.

Unlike all other known plastid sources of long-term-retention slugs, *Vaucheria litorea* (Fig. 1), the sole prey of *E. chlorotica,* is not a chlorophyte green alga but a heterokont yellow-green alga, with plastids derived from the red algal lineage through secondary endosymbiosis (Cruz *et al.*, 2013) (Fig. 1B). The plastome of *V. litorea* possesses the three important genes (de Vries *et al.*, 2013). Furthermore, the plastid-encoded FtsH of *V. litorea* has been shown to carry the critical metalloprotease domain that is not encoded in the *ftsH* genes of other prey algae of long-term-retention slugs (Christa *et al.*, 2018). Here, we show that isolated plastids of *V. litorea* (Fig. 1C) maintain highly specific transcription of their genes and exhibit adequate genetic autonomy in their capability to recover from light induced damage of PSII, that is, photoinhibition. Using the thoroughly studied species spinach as a reference, we also estimated reactive oxygen species (ROS) production in the thylakoid membranes of *V. litorea*, with special focus on the main ROS produced by PSII, singlet oxygen (^1^O_2_). While our results highlight the importance of terminal electron acceptors downstream of photosystem I (PSI) in limiting ROS production, we show that PSII and possibly also the ROS detoxification systems of *V. litorea* are fine-tuned to decrease the yield of the highly reactive ^1^O_2_. The consequences of our findings regarding light-induced damage to and longevity of the plastids inside photosynthetic sea slugs are discussed in detail.

## Materials and methods

### Organisms and culture conditions

To estimate the photosynthetic characteristics of the yellow-green alga *V. litorea,* we chose spinach, one of the most studied organisms in the field of photosynthesis research, as a reference species for the experiments. *Spinacia oleracea* L. Matador (Nelson Garden, Tingsryd, Sweden), and *V. litorea* C. Agardh 1823 (SCCAP K-0379) were grown in SGC 120 growth chambers (Weiss Technik UK, Loughborough, UK) under 8 h/12 h and 12 h/12 h light/dark cycles, respectively. The growth light (Master TL-D 36 W/840; Philips, Amsterdam, The Netherlands) photosynthetic photon flux density (PPFD) was set to 40 µmol m^–2^s^–1^ for both species. Temperature was maintained at 22 °C for spinach and 17 °C for *V. litorea.* Spinach plants used in the experiments were approximately 2 months old. *V. litorea* was grown in 500 ml flasks in f/2 culture medium (modified from Guillard and Ryther, 1962) made up in 1% (m/v) artificial sea water (Sea Salt Classic, Tropic Marin, Wartenberg, Germany). The *V. litorea* cultures were routinely refreshed by separating 1–4 g of inoculate into new flasks, and cultures used in the experiments were 1–2 weeks old. Nuclei of *V. litorea* were stained for microscopy with Hoechst 33342 (Thermo Scientific, Waltham, MA, USA) using standard protocols. *In vivo* transmission electron microscope (TEM) images were taken after freeze-etch fixation.

Unfortunately, we did not have access to the sea slug *E. chlorotica* to assess the functionality of *V. litorea* plastids inside this species. Instead, we used *Elysia timida,* another model species of photosynthetic sea slug capable of long-term plastid retention. *Elysia timida* feeds on and steals plastids from the green alga *Acetabularia acetabulum*; both species were routinely maintained as described previously (Schmitt *et al.*, 2014; Havurinne and Tyystjärvi, 2020). We only used *E. timida* individuals in the experiments, *A. acetabulum* was grown to be used as a source of the plastids for this slug, and the use of *E. timida* is specifically indicated when pertinent.

### Gene expression of isolated *V. litorea* plastids

Plastid isolation from *V. litorea* was performed based on Green *et al.* (2005). Briefly, filaments were cut into small pieces, resuspended in 40 ml of isolation buffer (see Table 1), and homogenized with an ULTRA-TURRAX® (IKA, Staufen, Germany) using four short bursts at 8000 rpm. The homogenate was filtered twice through a layer of Miracloth (Calbiochem, Darmstadt, Germany) and then centrifuged at 1900 *g* for 5 min, and the pellet was resuspended in 1 ml of isolation buffer. Percoll solution containing 0.25 M sucrose was diluted to a 75% and a 30% solution with 1× TE buffer containing 0.25 M sucrose. The sample was layered between the two dilutions and the assemblage was centrifuged at 3500 *g* for 20 min in a swing-out rotor with no deceleration. Intact plastids were collected from the interphase and washed twice by centrifugation at 2200 *g* for 3 min with isolation buffer lacking BSA. All steps were carried out at 4 °C in the dark. TEM imaging of the plastids was done after fixing the samples using glutaraldehyde and cryo-fixation followed by freeze substitution.

**Table 1.** Buffer solutions used in sample preparation and measurements

Identifier	Composition	Used in
Plastid isolation buffer	0.2% BSA, 1 mM EDTA, 50mM HEPES-KOH, pH 7.6, 1 mM MgCl_2_, 330 mM sorbitol	Plastid isolation, *in organello* gene expression
Thylakoid isolation buffer	1% BSA, 1 mM EDTA, 1 mM glycine betaine, 40 mM HEPES-KOH, pH 7.4, 10 mM MgCl_2_, 0.3 M sorbitol	Thylakoid isolation
Osmotic shock buffer	10 mM HEPES-KOH, pH 7.4, 10 mM MgCl_2_, 5 mM sorbitol	Thylakoid isolation
Thylakoid storage buffer	10 mM HEPES-KOH, pH 7.4, 10 mM MgCl_2_, 5 mM NaCl, 500 mM sorbitol	Thylakoid isolation, EPR
Photosystem measuring buffer	1 M glycine betaine, 40 mM HEPES-KOH, pH 7.4, 1 mM KH_2_PO_4_, 5 mM MgCl_2_, 5 mM NaCl, 5 mM NH_4_Cl, 330 mM sorbitol	Thermoluminescence, flash oxygen evolution, fluorescence decay kinetics, ^1^O_2_ production
Photoinhibition buffer	1 M glycine betaine, 40 mM HEPES-KOH, pH 7.4, 5 mM MgCl_2_, 5 mM NaCl, 330 mM sorbitol	*In vitro* photoinhibition treatments, *in vitro* P_700_^+^ measurements
PSI measuring buffer	Photosystem measuring buffer + 0.3 mM 2,6-dichlorophenolindophenol (DCPIP), 0.01 mM 3-(3,4-dichlorophenyl)-1,1-dimethylurea (DCMU), 0.12 mM methyl viologen, 32 mM Na ascorbate, 0.6 mM NaN_3_	Polarographic PSI activity measurements (oxygen consumption)
PSII measuring buffer	Photosystem measuring buffer + 0.5 mM 2,6-dichloro-1,4-benzoquinone (DCBQ), 0.5 mM hexacyanoferrate(III)	Polarographic PSII activity measurements (oxygen evolution)

Plastids were kept in isolation buffer for 7 days in routine culture conditions. RNA was isolated at different time points using a Spectrum™ Plant Total RNA Kit (Sigma-Aldrich, St. Louis, MO, USA). Aliquots with 50 ng RNA were subjected to treatment with DNAse (Thermo Scientific), and treated aliquots amounting to 10 ng RNA were used for cDNA synthesis (iScript™ cDNA Synthesis Kit, BioRad, Hercules, CA, USA). Quantitative real-time PCR (qPCR) was carried out using a StepOnePlus (Applied Biosystems, Foster City, CA, USA) and reagents from BioRad. The primers used in the qPCR were designed using Primer3 (http://frodo.wi.mit.edu/primer3); the primer sequences are listed in Supplementary Table S1. Every reaction was done with technical triplicates using the following thermal cycle: 95 °C for 2 min, then 40 cycles of 95 °C for 5 s and 60 °C for 30 s. At the end of each qPCR run, the temperature was increased in a stepwise manner from 60 °C to 95 °C to obtain the melting curves. The results were analyzed using the ∆∆Ct method (Pfaffl, 2001), in which the qPCR data were normalized to reference genes and time point 0 (immediately after plastid isolation). The decrease in the relative amounts of transcripts during incubation of isolated chloroplasts was estimated from the number of qPCR cycles required to reach the threshold level of the signal, and these data were used to select *rbcL* and *psaA* as the reference genes.

### *In vivo* photoinhibition

The capacity to recover from photoinhibition was tested in spinach leaves and *V. litorea* cells in the presence of cycloheximide (CHI), a cytosolic translation inhibitor. Spinach leaf petioles were submerged in water containing 1 mM CHI and incubated for 24 h in the dark. The incubation was identical for *V. litorea* cells, except that the cells were fully submerged in f/2 medium supplemented with 1 mM CHI. Control samples were treated identically without CHI. The samples were then exposed to white light (PPFD 2000 µmol m^–2^ s^–1^) for 60 min and subsequently placed in the dark for 60 min and thereafter in low light (PPFD 10 µmol m^–2^ s^–1^) to recover for 180 min. The temperature was maintained at the growth temperatures of both species using a combination of a thermostat-controlled surface and fans. The petioles of spinach leaves were submerged in water (with or without CHI) during the experiments. Cell clusters of *V. litorea* were placed on top of the thermostat-controlled surface on a paper towel moistened thoroughly with f/2 medium (with or without CHI). PSII activity was estimated by measuring the ratio of variable to maximum fluorescence (F_V_/F_M_) (Genty *et al.*, 1989) with a PAM-2000 (Walz, Effeltrich, Germany) fluorometer. During the light treatments, F_V_/F_M_ was measured from samples that were dark acclimated for <5 min, except for the final time point, where the samples were dark acclimated for 20 min. The light source used for all high-light treatments described in this study was an Artificial Sunlight Module (SLHolland, Breda, The Netherlands).

Membrane proteins were isolated at the following time points: 0, 60, 120, and 300 min (Fig. 3C-F). The same area in which F_V_/F_M_ was measured (~1 cm^2^) was cut out of the leaves/algal clusters and placed in a 1 ml Dounce tissue grinder (DWK Life Sciences, Millville, NJ, USA) filled with 0.5 ml of osmotic shock buffer (Table 1) and ground thoroughly. The homogenate was filtered through one layer of Miracloth and centrifuged at 5000 *g* for 5 min. The pellet containing the membrane protein fraction was resuspended in 50 µl of thylakoid storage buffer. The samples were stored at –80 °C until use. Membrane protein samples containing 1 µg total Chl were solubilized and separated by electrophoresis on a 10% SDS-polyacrylamide gel using Next Gel solutions and buffers (VWR, Radnor, PA, USA). Proteins were transferred to Immobilon-P polyvinylidene difluoride membranes (MilliporeSigma, Burlington, MA, USA). FtsH was immunodetected using antibodies raised against *Arabidopsis thaliana* FtsH5, reactive with the highly homologous proteins FtsH1 and FtsH5, or FtsH2, reactive with FtsH2 and FtsH8 (Agrisera, Vännäs, Sweden). Western blots were imaged using goat anti-rabbit IgG (H+L) alkaline phosphatase conjugate (Life Technologies, Carlsbad, CA, USA) and CDP-star Chemiluminescence Reagent (Perkin-Elmer, Waltham, MA, USA). Protein bands were quantified with Fiji (Schindelin *et al.*, 2012).

Experiments with *E. timida* were performed on freshly fed individuals. Slugs were kept in the dark overnight in both the absence and presence of 10 mg ml^–1^ lincomycin in 3.7% artificial sea water and then exposed to high light (PPFD 2000 µmol m^–2^ s^–1^) in wells of a 24-well plate filled with artificial sea water for 40 min. The temperature was maintained at 23 °C throughout the treatment. The slugs were then placed in low light (PPFD <20 µmol m^-2^ s^-1^) overnight, in their growth conditions, to recover. F_V_/F_M_ was measured with a PAM-2000 fluorometer after a minimum 20 min dark period as described previously (Havurinne and Tyystjärvi, 2020).

### Isolation of functional thylakoids for *in vitro* experiments

Functional thylakoids were isolated as described previously (Hakala *et al.*, 2005) after 24 h incubation in the dark. One spinach leaf per isolation was ground in a mortar in thylakoid isolation buffer (Table 1). The homogenate was filtered through a layer of Miracloth and pelleted by centrifugation at 5000 *g* for 5 min. The pellet was resuspended in osmotic shock buffer and then centrifuged at 5000 *g* for 5 min, and the resulting pellet was resuspended in thylakoid storage buffer. Thylakoid isolation from *V. litorea* was performed using the same procedure, by grinding 2–5 g of fresh cell mass per isolation. The cell mass was briefly dried between paper towels before grinding. The protein concentrations of the thylakoid suspensions were determined with a DC™ Protein Assay (Bio-Rad, Hercules, CA, USA). Thylakoids used in functional experiments were kept on ice in the dark and always used within a few hours of isolation.

### Pigment analysis

Concentrations of Chl were determined spectrophotometrically (Shimadzu UV-1900 spectrophotometer; Kyoto, Japan) in 90% acetone using extinction coefficients for Chls *a* and *b* with spinach, and Chls *a* and *c1*+*c2* with *V. litorea* (Jeffrey and Humphrey, 1975). The 400–750 nm absorption spectra of the pigments from the thylakoids of both species were measured after *N*,*N*-dimethylformamide (DMF) extraction. For HPLC analysis, pigments were extracted from 50 µl of isolated spinach and *V. litorea* thylakoids with 500 µl of pure methanol. The extracts were then centrifuged at 12 000 *g* for 15 min and the supernatant was collected into a microcentrifuge tube. This procedure was repeated three times. Finally, the extract was filtered with a 0.2 µm syringe filter. Photosynthetic pigments were separated by HPLC according to (Gilmore and Yamamoto, 1991) with some modifications, using a reverse phase C18 column (LiChroCART 125-4; Hewlett Packard, Palo Alto, CA, USA) in a series 1100 HPLC device with diode array and fluorescence detector (Agilent Technologies, Palo Alto, CA, USA). Buffer A consisted of acetonitrile, methanol, and 0.1 M Tris–HCl solution, pH 8.0 (72:8:3, v/v), and buffer B consisted of methanol and hexane (4:1, v/v). The constant flow rate was 0.75 ml min^–1^ and the temperature of the column was maintained at 15 °C. The program started with an isocratic run with buffer A for 4 min, followed by a linear gradient for 15 min from 0% buffer B to 100% buffer B. The isocratic run of buffer B lasted 26 min, and was followed by a linear gradient for 2 min from 100% buffer B to 100% buffer A. The column was re-equilibrated between samples for a minimum of 12 min with buffer A. Pigments were detected via absorption at 440 nm, and α-tocopherol was detected by fluorescence (I_ex_=295 nm, I_em_=340 nm). Pigment standards were obtained from DHI Lab Products (Hørsholm, Denmark).

### Photosystem stoichiometry

Photosystem stoichiometry was measured from thylakoid membranes with an electron paramagnetic resonance (EPR) spectroscope (Miniscope MS5000, Magnettech GmbH, Berlin, Germany) as described previously (Tiwari *et al.*, 2016; Nikkanen *et al.*, 2019). EPR spectra originating from the oxidized tyrosine-D residue of PSII (Tyr_D_^+^) and reaction center Chl of PSI (P_700_^+^) of concentrated thylakoid samples (2000 µg Chl ml^–1^ in storage buffer) were measured in a magnetic field ranging from 328.96 mT to 343.96 mT during illumination (PPFD 4000 µmol m^–2^ s^–1^) (Lightningcure LC8, Hamamatsu Photonics, Hamamatsu City, Japan) and after a subsequent 5 min dark period in the absence and presence of 50 µM 3-(3,4-dichlorophenyl)-1,1-dimethylurea (DCMU). The dark-stable Tyr_D_^+^ EPR signal (PSII signal), measured after the post-illumination period in the absence of DCMU, and the P_700_^+^ (PSI signal), measured during illumination in the presence of DCMU, were double integrated to determine photosystem stoichiometry.

### *In vitro* photoinhibition

For *in vitro* photoinhibition experiments, thylakoids were diluted to a total Chl concentration of 100 µg ml^–1^ in photoinhibition buffer (Table 1), and a 1 ml sample was loaded into a glass beaker submerged in a water bath kept at 22 °C. The samples were exposed to white light (PPFD 1000 µmol m^–2^ s^–1^) and mixed with a magnet during the 60 min treatments. Aliquots were taken at set intervals to determine the activity of PSI or PSII using a Clark-type oxygen electrode (Hansatech Instruments, King’s Lynn, UK). The sample concentration in the activity measurements was 20 µg total Chl ml^–1^ in 0.5 ml of PSI or PSII measuring buffer (Table 1). PSI activity was measured as oxygen consumption, whereas PSII activity was measured as oxygen evolution. Both activities were measured at 22 °C in strong light (PPFD 3200 µmol m^–2^ s^–1^) from a slide projector. The rate constant of PSII photoinhibition (*k*_PI_) was obtained by fitting the loss of oxygen evolution to a first-order reaction equation with Sigmaplot 13.0 (Systat Software, San Jose, CA, USA), followed by dark correction, that is, subtraction of the dark inactivation rate constant from the initial *k*_PI_.

Lipid peroxidation was measured by detecting malondialdehyde (MDA) formation (Heath and Packer, 1968). An aliquot of 0.4 ml of thylakoid suspension was mixed with 1 ml of 20% trichloroacetic acid containing 0.5% thiobarbituric acid, incubated at 80 °C for 30 min, and cooled on ice for 5 min. Excess precipitate was pelleted by centrifugation at 13 500 *g* for 5 min, and the difference in absorbance between 532 nm and 600 nm (Abs_532-600_) was measured as an indicator of the relative amount of MDA in the samples. Protein oxidation was determined by detecting protein carbonylation with an Oxyblot™ Protein Oxidation Detection Kit (MilliporeSigma, Burlington, MA, USA). Aliquots of thylakoid suspension amounting to a protein content of 45 µg were taken at set time points and 10 mM dithiothreitol was used to prevent further protein carbonylation. The samples were prepared according to the manufacturer’s instructions and proteins were separated in 10% Next Gel SDS-PAGE (VWR). Carbonylated proteins were detected with Immobilon Western Chemiluminescent HRP Substrate (MilliporeSigma).

The maximum oxidation of P_700_ (P_M_) was estimated in an additional experiment. Thylakoids equivalent to 25 µg Chl in 50 µl of photoinhibition buffer were pipetted on to a Whatman filter paper (grade 597; Cytiva, Marlborough, MA, USA). The filter was placed inside the lid of a plastic Petri dish, and the bottom of the Petri dish was placed on top of the lid. Photoinhibition buffer was added to the sample through the small openings on the sides of the assemblage. The thylakoids were then illuminated with high light (PPFD 1000 µmol m^–2^ s^–1^) and the temperature was maintained at 22 °C using a thermostat-controlled surface. F_V_/F_M_ and P_M_ were measured using a 700 ms high-light pulse (PPFD 10 000 µmol m^–2^ s^–1^) at set intervals with a Dual-PAM 100 (Walz) (Schreiber, 1986; Schreiber and Klughammer, 2008). The high-light-treated samples were dark acclimated for <5 min before the measurements were made.

### ^1^O_2_ measurements

^1^O_2_ was measured in thylakoids diluted to 100 µg total Chl ml^–1^ in 0.3 ml of photosystem measuring buffer (Table 1), using the highly selective histidine method described previously (Telfer *et al.*, 1994; Rehman *et al.*, 2013). Briefly, the imidazole ring of histidine reacts efficiently with ^1^O_2_ (rate constant 5×10^7^ M^–1^s^–1^) (Bisby *et al.*, 1999) and therefore, in the presence of histidine, ^1^O_2_ is rapidly consumed in this reaction, which leads to a decrease in the overall oxygen concentration. Continuously stirred thylakoid samples were exposed to high light (PPFD 3200 µmol m^–2^ s^–1^) from a slide projector at 22 °C in the presence and absence of 20 mM histidine. Oxygen consumption was measured for 60 s using an oxygen electrode (Hansatech), and the difference in the oxygen consumption rates in the presence and absence of histidine was taken as an indicator of ^1^O_2_ production. PSII electron transfer activity [H_2_O to 2,6-dichloro-1,4-benzoquinone (DCBQ)] in the same conditions was 124.7 (SE ±15.4) and 128.4 (SE ±10.7) µmol O_2_ mg Chl^–1^ h^–1^ in spinach and *V. litorea* samples, respectively, containing 20 µg Chl ml^–1^.

### PSII charge recombination measurements

Flash-induced oxygen evolution was recorded at room temperature using a Joliot-type bare platinum oxygen electrode (PSI, Brno, Czech Republic) (Joliot and Joliot, 1968) with thylakoids diluted in photosystem measuring buffer to 50 µg Chl ml^–1^ and supplemented with 50 mM KCl, essentially as described in Antal *et al.* (2009). A 200 µl sample was pipetted on to the electrode and kept in the dark for 10 min before the measurements were made. The samples were then exposed to a flash train consisting of 15 single-turnover flashes (4 ns per pulse) at 1 s intervals, provided by a 532 nm Nd:YAG laser (Minilite, Continuum, San Jose, CA, USA). Charge recombination within PSII was probed by exposing the samples to a preflash and different dark periods between the preflash and the flash train used for recording the oxygen traces.

The decay of Chl *a* fluorescence yield after a 30 µs single-turnover flash (maximum PPFD 100 000 µmol m^-2^ s^-1^) was measured at room temperature in 1 ml samples of thylakoids using a FL200/PS fluorometer (PSI). Measurement length was 120 s, and eight data points per decade were recorded (two in the presence of DCMU), using the formula P_1_10^(n/N)^, where P_1_ is the starting time point and N is the number of data points per decade, to determine the nth time point. The first data point was recorded 150 µs after the flash. Single-turnover flash and measuring beam voltages were set to 100% and 60% of the maximum, respectively. The samples were diluted in photosystem measuring buffer to a total Chl concentration of 20 µg ml^–1^. A set of samples was poisoned with 20 µM DCMU to block electron transfer at the reducing side of PSII.

Thermoluminescence of thylakoids was measured using a custom setup (Tyystjärvi *et al.*, 2009). Thylakoids were diluted to a total Chl concentration of 100 µg ml^–1^ in photosystem measuring buffer in the presence and absence of 20 µM DCMU, and a volume of 100 µl was pipetted on to a filter paper disk that was placed inside the cuvette of the measuring apparatus. The samples were dark acclimated for 5 min before the onset of cooling to –20 °C by a Peltier element (TB-127-1,0-0,8, Kryotherm, Carson City, NV, USA). The samples were then exposed to a flash (E=1 J) from a FX-200 xenon lamp (EGandG, Gaithersburg, MD, USA) and heated to 60 °C at a rate of 0.47 °C s^–1^ while simultaneously recording luminescence emission.

### *In vivo* P_700_ redox kinetics

Redox kinetics of P_700_ were measured as described by Shimakawa *et al.* (2019) using a Dual-PAM 100 (Walz). Spinach plants and *V. litorea* cells were kept in darkness for at least 2 h before the measurements were made. Anaerobic conditions were obtained using a custom cuvette described in Havurinne and Tyystjärvi (2020). For spinach leaf cutouts, the cuvette was flushed with nitrogen. A combination of glucose oxidase (8 units ml^–1^), glucose (6 mM), and catalase (800 units ml^–1^) in f/2 culture medium was used to create anaerobic conditions for *V. litorea* cells. All samples were treated with 15 s of far-red light (photon flux density 120 µmol m^–2^ s^–1^) and a subsequent period of darkness of 25 s before firing a high-light pulse (780 ms, PPFD 10 000 µmol m^–2^ s^–1^).

## Results

### Isolated *V. litorea* plastids maintain regulated gene expression

**Fig. 2.**
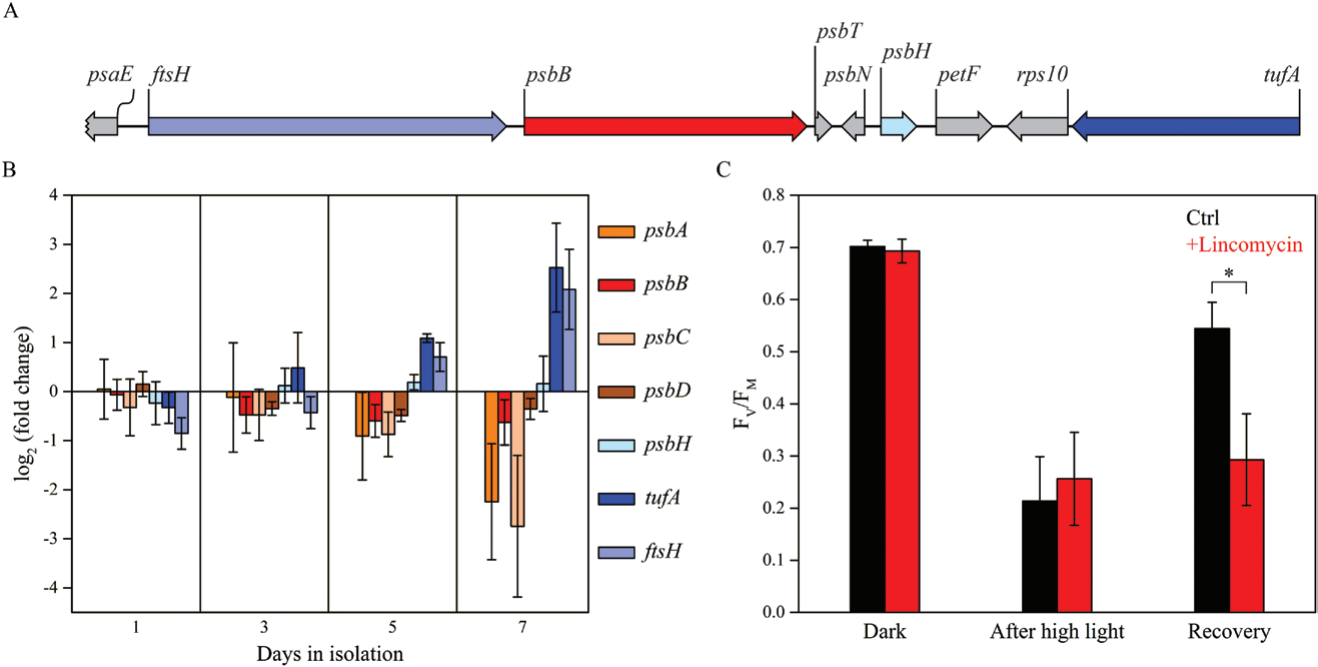
Transcription of plastid-encoded genes in isolated *V. litorea* plastids and the autonomy of kleptoplasts inside the sea slug *E. timida*. (A) Orientation of specific genes inspected in (B) in the *V. litorea* plastid genome. (B) Amounts of transcripts of selected genes during a period of 7 days in isolation buffer. Each transcript has been compared to the amount measured immediately after plastid isolation and normalized to *rbcL* and *psaA* transcripts. (C) Maximum quantum yield of PSII photochemistry (F_V_/F_M_) measured at different time points of the photoinhibition treatment (40 min, PPFD 2000 µmol m^–2^s^–1^) and after overnight recovery (PPFD <20 µmol m^–2^ s^–1^) in individual *E. timida* slugs in the absence and presence of lincomycin. The data in (B, C) are means ±SD from three and four biological replicates, respectively. The asterisk indicates a statistically significant difference between the two groups (*P<0.005, Welch’s *t*-test).

For the analysis of plastid gene expression, laboratory-isolated *V. litorea* plastids were incubated for 7 days in isolation. qPCR was done for nine transcripts of mainly photosynthesis-related genes, representing 6.5% of the 139 protein-coding genes of the *V. litorea* plastome (Rumpho *et al.*, 2008). The orientations of the selected genes in the plastome are shown in Fig. 2A. The *rbcL* and *psaA* genes were chosen as reference genes on the basis of the number of PCR cycles required for the genes to reach the threshold value during the 7-day incubation. This number increased from 25.3 to 30.1 for *rbcL* and from 24.7 to 29.6 for *psaA*, indicating that the number of the transcripts of these two genes decreased in a similar manner in isolation (Supplementary Fig. S1). More importantly, the decrease in the transcripts during the first 3 days was roughly similar to the decay of intact chloroplasts of *V. litorea* in isolation (Green *et al.*, 2005). These data suggest that *rbcL* and *psaA* were stably expressed in the isolated chloroplasts that remained intact in isolation.

The qPCR data show that laboratory-isolated *V. litorea* plastids exhibited differentially regulated gene expression even after 7 days in isolation (Fig. 2A). The PSII core subunit genes *psbA*, *psbB*, *psbC*, and *psbD* were down-regulated after day 3 of the isolation period; *psbB* and *psbD*, encoding the CP47 and D2 proteins of PSII, reached a stationary level of expression after 5 days, whereas the genes encoding the PSII proteins CP43 (*psbC*) and D1 (*psbA*) were down-regulated to the greatest extent (Fig. 2B). The main protein of PSII targeted for degradation after photoinhibition is D1, whereas release of CP43 from the PSII core has been suggested to precede D1 degradation in higher plants (Aro *et al.*, 2005). One gene, *psbH*, encoding a small PSII subunit involved in proper PSII assembly in cyanobacteria (Komenda *et al.*, 2005), exhibited similar transcript levels to the control genes *rbcL* and *psaA* throughout the isolation period. The relative transcript amounts of *ftsH* and *tufA*, encoding the maintenance protease FtsH and the translation elongation factor EF-Tu, followed an upward trajectory throughout the experiment relative to the two control genes (Fig. 2B).

We also tested the genetic autonomy of plastids in the photosynthetic sea slug model E*. timida.* This slug species is capable of long-term retention of plastids, and feeds on the green alga *A. acetabulum*, not *V. litorea*. Subjecting the slugs to high light for 40 min resulted in a drastic decrease in PSII photochemistry (F_V_/F_M_), but the kleptoplasts inside the slugs were capable of restoring PSII activity back to 78% of the initial level during a 20 h recovery period. Subjecting the slugs to treatment with lincomycin, a plastid-specific translation inhibitor (Mulo *et al.*, 2003), however, almost completely prevented the recovery of F_V_/F_M_ (Fig. 2C). Surprisingly, lincomycin-treated slugs showed similar F_V_/F_M_ values after the high-light treatment to those of slugs illuminated in the absence of lincomycin. This could be partially due to the increased mucus excretion of the lincomycin-treated slugs, and their tendency to curl up next to the edges of the well more tightly than the control slugs, which may have helped them avoid the strong light. Similar concentrations of lincomycin have been used in previous studies on photosynthetic sea slugs (Christa *et al.*, 2018). In our conditions, the slugs usually survived the lincomycin treatment, and after the experiments they were returned to their normal growth conditions to feed on plenty of algae. Observation of the slugs after the experiment did not reveal major signs of detrimental effect from the lincomycin treatment.

### FtsH translation is enhanced in functionally isolated plastids of *V. litorea* during recovery from photoinhibition

**Fig. 3.**
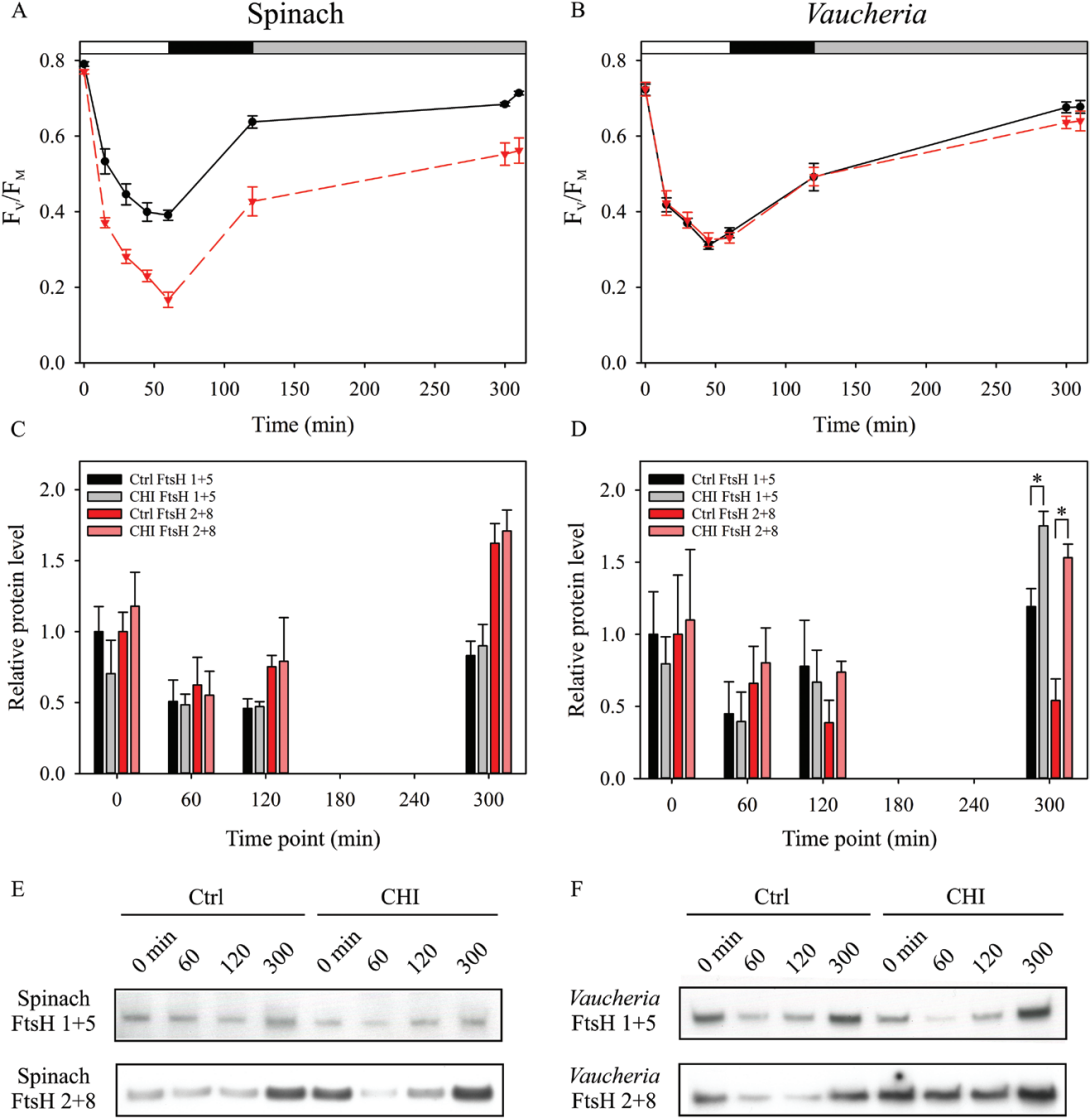
*V. litorea* recovers from photoinhibition of PSII in the presence of CHI, a cytosolic translation inhibitor, and exhibits strong expressions of FtsH during recovery. (A, B) Quantum yield of PSII photochemistry (F_V_/F_M_) during photoinhibition treatment and subsequent recovery of (A) spinach and (B) *V. litorea* in the absence (black) and presence (red) of CHI. The 0 min time point was measured before the onset of high-light treatment, the 60 min time point after the high-light treatment (PPFD 2000 µmol m^–2^s^–1^), the 120 min time point after subsequent dark recovery, the 300 min time point after recovery in dim light (10 µmol m^–2^s^–1^), and the final time point at 310 min after an additional 10 min dark acclimation. The white, black, and gray bars at the top indicate the high-light treatment, dark, and dim light periods, respectively. (C, D) Relative levels of FtsH in (C) spinach and (D) *V. litorea* during the experiment, as probed by antibodies raised against *A. thaliana* FtsH5 (FtsH 1 + 5; black and grey bars for the control and CHI treatments, respectively) and FtsH2 (FtsH 2 + 8; red and light red bars for the control and CHI treatments, respectively). The light treatment regime up to 300 min was the same as in (A, B). Significant differences between treatments are indicated by asterisks (*P<0.05, Welch’s *t*-test, *n*=3). (E, F) Representative FtsH Western blots from (E) spinach and (F) *V. litorea* used for protein quantification in (C, D). All data in (A–D) represent means ±SE from at least three independent biological replicates.

Treating spinach leaves with CHI, a cytosolic translation inhibitor, resulted in faster loss of PSII activity in high light (Fig. 3A). In addition, PSII repair was impaired by CHI in spinach. By contrast, *V. litorea* showed almost no effect of CHI during the same photoinhibition and recovery treatment (Fig. 3B). Using two different antibodies against FtsH (FtsH 1 + 5 and FtsH 2 + 8), we tested the possible involvement of plastid-encoded FtsH of *V. litorea* in the unaffected PSII photochemistry in CHI-treated samples. There were no differences in the relative protein levels of FtsH between control and CHI-treated spinach during the experiment (Fig. 3C). Genes encoding FtsH reside in the nucleus in spinach, and our results suggest that the CHI treatment did not inhibit cytosolic translation in the leaves entirely, although *de novo* synthesis of proteins could not be tested by radiolabeling experiments. In *V. litorea,* CHI treatment increased the FtsH levels towards the end of the experiment (Fig. 3D). This result suggests that not only is the expression of plastome genes active in functionally isolated plastids of *V. litorea*, but the translation of specific genes such as *ftsH* can be enhanced when the plastids are deprived of normal cytosolic governance.

### Thylakoids of *V. litorea* exhibit moderate photoinhibition of PSII and elevated ROS damage, but produce little ^1^O_2_

**Fig. 4.**
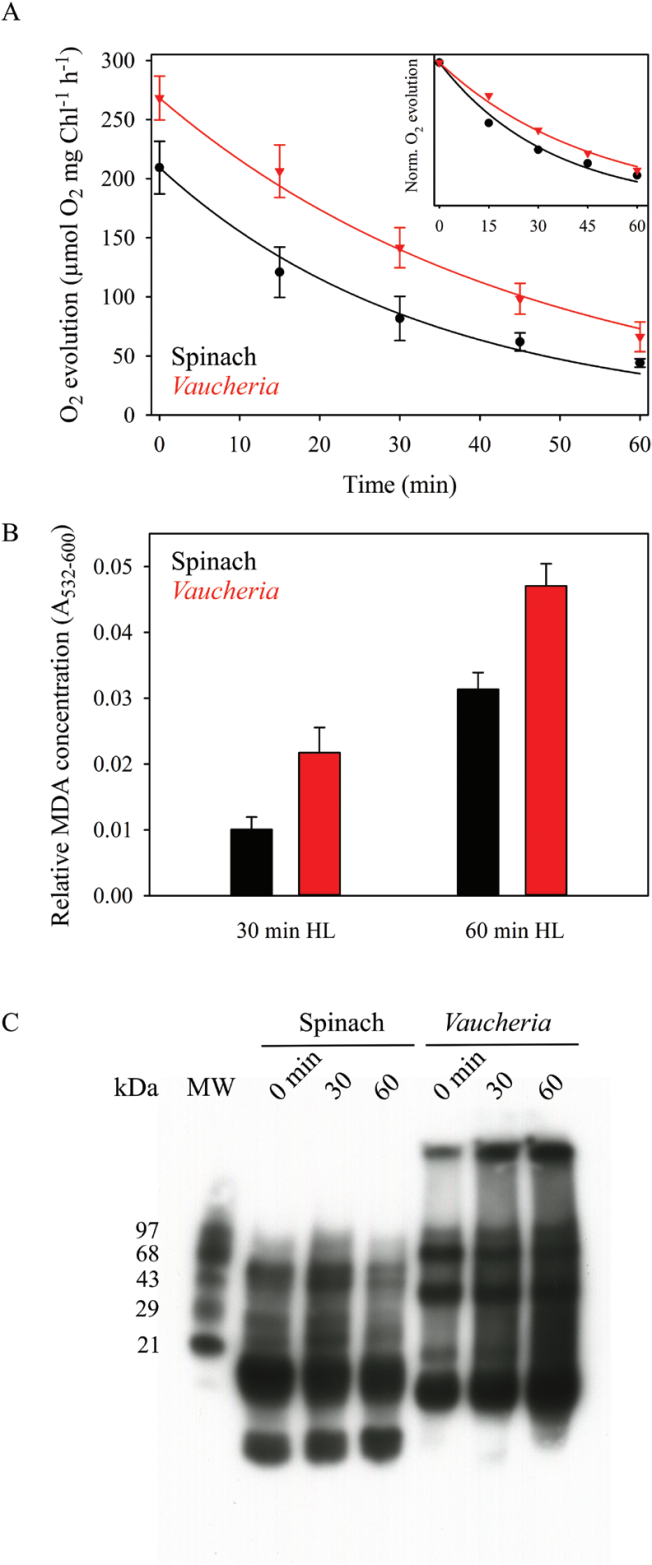
*In vitro* photoinhibition of PSII and ROS production in spinach and *V. litorea* thylakoids in high light. (A) Photoinhibition of PSII in high light (PPFD 1000 µmol m^–2^s^–1^) as estimated by oxygen evolution. The curves show the best fit to a first-order reaction in spinach and *V. litorea*. Data normalized to the initial oxygen evolution rates are shown in the inset to facilitate comparison. Dark control experiments, shown in Supplementary Fig. S3, indicated a 4.9% (SE ±3.6, *n*=3) and 27.5% (SE ±6.7, *n*=3) loss of PSII activity after 60 min in the dark for spinach and *V. litorea*, respectively. (B) Lipid peroxidation after 30 min and 60 min of high-light treatment in spinach and *V. litorea*, as indicated by MDA formation. MDA formed during dark control treatments was subtracted from the high-light treatment data. (C) A representative Oxyblot™ assay of protein carbonylation during the high-light treatment. The data in (A, B) represent the means ±SD from a minimum of three biological replicates.

**Fig. 5.**
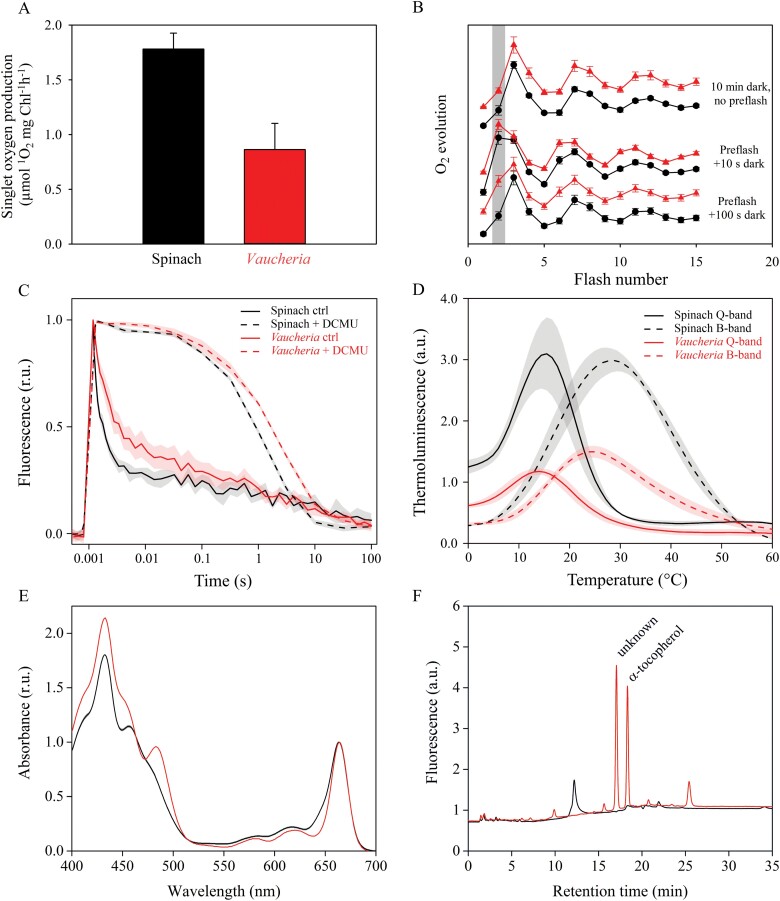
Thylakoids of *V. litorea* produce little ^1^O_2_ and exhibit slow charge recombination of PSII and abundant antioxidants. (A) ^1^O_2_ production in spinach and *V. litorea* thylakoid membranes. (B) Flash oxygen evolution after different preflash treatments in spinach (black) and *V. litorea* (red) thylakoids. The gray bar highlights the oxygen yield instigated by the second flash, an indicator of charge recombination reactions taking place during the dark period between a preflash and the measuring flash sequence. Oxygen traces were double normalized to the first (zero level) and third flash and shifted in the Y-axis direction for clarity. (C) Chl fluorescence decay kinetics after a single-turnover light pulse in untreated (ctrl) and DCMU-poisoned thylakoids, double normalized to zero level before the onset of the pulse and maximum fluorescence measured 150 µs after the pulse. (D) Q and B bands of thermoluminescence, measured in the presence and absence of DCMU. (E) Absorption spectra of spinach and *V. litorea* thylakoid pigments after DMF extraction, normalized to their respective Chl *a* absorption peaks at 664 nm. (F) HPLC chromatogram from spinach and *V. litorea* thylakoid samples extracted in pure methanol, showing the α-tocopherol fluorescence peak (I_ex_=295 nm, I_em_=340 nm), as indicated. All data in (A–C) represent means ±SE from at least three biological replicates, whereas the data in (D, E) are from three replicates obtained from pooled thylakoid batches isolated from three plants/algae flasks. The chromatograms shown in (F) are representative graphs of the data obtained from triplicate runs of similarly pooled thylakoids as in (D, E).

Basic photosynthetic parameters of isolated thylakoids from spinach and *V. litorea* are shown in Table 2. Photoinhibition of PSII during a 60 min high-light treatment of isolated thylakoids proceeded according to first-order reaction kinetics (Tyystjärvi and Aro, 1996) in both species (Fig. 4A). However, spinach thylakoids were more susceptible to damage, as indicated by the larger rate constant of dark-corrected PSII photoinhibition (*k*_PI_) (Table 2). General oxidative stress assays of lipids and proteins of the thylakoid membranes exposed to high light showed more ROS damage in *V. litorea* than in spinach thylakoids during the treatment (Fig. 4B, C). Measurements of the production of ^1^O_2_, the main ROS produced by PSII (Krieger-Liszkay, 2005; Pospíšil, 2012), by isolated thylakoids showed that the amount of ^1^O_2_ from illuminated *V. litorea* thylakoids was only half of that observed for spinach thylakoids (Fig. 5A). This finding suggests that the main ROS causing the *in vitro* oxidative damage to lipids and proteins (Fig. 4B, C) in *V. litorea* are likely ROS produced on the PSI side of the electron transfer chain, such as superoxide anion radical, hydrogen peroxide, or hydroxyl radical. The individual contributions of specific ROS to the oxidative damage shown in Fig. 4 could not be estimated from the current data.

**Table 2.** Photosynthesis-related parameters of isolated spinach and *V. litorea* thylakoid membranes

Organism	PSI/PSII	PSII activity (H_2_O to DCBQ; µmol O_2_ evolved mg Chl^–1^ h^–1^)	PSI activity (DCPIP to methyl viologen; µmol O_2_ consumed mg Chl^–1^ h^–1^)	*k*_PI_ (min^–1^)
Spinach	2.438±0.100	200.12±11.53	758.22±77.14	0.0289±0.002
*V. litorea*	2.343±0.090	244.54±15.71	797.36±93.73	0.0148±0.001

The EPR spectra used for estimating the PSI/PSII ratio are shown in Supplementary Fig. S2. The indicated PSII and PSI activities are averages from all initial activity measurements of untreated control samples discussed in this publication. The *k*_PI_ value was determined from first-order reaction fits of the photoinhibition data in Fig. 4A, and corrected by subtracting the first-order rate constant of PSII inhibition in the dark (Supplementary Fig. S3). All values are the means ±SE from a minimum of three biological replicates.

### Thylakoids of *V. litorea* produce little ^1^O_2_, likely due to slow PSII charge recombination and efficient antioxidants

We probed charge recombination reactions within PSII by using three different methods to investigate the role of PSII in the low ^1^O_2_ yield in *V. litorea* thylakoids (Fig. 5A). First, we measured flash-induced oxygen evolution from isolated thylakoids of spinach and *V. litorea*. After 10 min dark acclimation, thylakoids from both species exhibited a typical pattern of oxygen evolution: the third flash caused the highest oxygen yield due to the predominance of the dark-stable S_1_ state of the oxygen-evolving complex (OEC), after which the oxygen yield oscillated with a period of four flashes until dampening occurred due to misses and charge recombination reactions (Fig. 5B, top pair of curves). A single-turnover preflash treatment makes S_2_ the predominant state. A 10 s dark period after the preflash treatment was not long enough to cause noticeable changes in the S-state distribution in either species, as can be seen from the middle pair of curves in Fig. 5B, where the second flash of the flash train causes the highest yield of oxygen. In spinach, 100 s of darkness after the preflash treatment resulted in nearly complete restoration of the original S-states, whereas in *V. litorea* the second flash still yielded a considerable amount of oxygen (Fig. 5B, bottom pair of curves). This is likely due to slow charge recombination between Q_B_^–^ and the S_2_ state of the OEC in *V. litorea* (Pham *et al.*, 2019). The modeled percentage S-state distributions of OEC from spinach and *V. litorea* after different dark times between the preflash and the flash train are shown in Supplementary Table S2.

Next, we measured the decay of Chl *a* fluorescence yield after a single-turnover flash from thylakoids in the absence and presence of the PSII electron transfer inhibitor DCMU. Fluorescence decay in the absence of DCMU reflects Q_A_^–^ reoxidation mainly by electron donation to Q_B_ and Q_B_^–^. In the presence of DCMU, fluorescence decay is indicative of Q_A_^–^ reoxidation through various charge recombination reactions (Mamedov *et al.*, 2000), some of which generate the harmful triplet P_680_ Chl through the intermediate P_680_^+^Pheo^–^ radical pair (Sane *et al.*, 2012). The decay of fluorescence yield was slower in *V. litorea* thylakoids than in spinach in both the absence and the presence of DCMU (Fig. 5C). In the absence of DCMU, the slower kinetics in *V. litorea* shows that electron transfer from Q_A_^–^ to Q_B_ is not as favorable as in spinach. The slow decay of fluorescence in the presence of DCMU indicates slow S_2_Q_A_^–^ charge recombination.

The Q and B thermoluminescence bands, obtained in the presence and absence of DCMU, respectively, were also measured from thylakoids. For a description of the interpretation of thermoluminescence data, see Tyystjärvi and Vass (2004) and Sane *et al.* (2012). Briefly, the thylakoid samples were dark acclimated for 5 min, cooled to –20 °C, flashed with a single-turnover xenon flash, and then heated to 60 °C at a constant rate. The luminescence emitted by the samples at different temperatures is proportional to the rate of the luminescence-producing charge recombination reactions between the S-states of the OEC and downstream electron acceptors, more specifically S_2_/Q_A_^–^ (Q band) and S_2,3_/Q_B_^–^ (B band). The Q- and B-band emission peaks in spinach were at 15 °C and 28 °C, respectively, whereas in *V. litorea* they were at 14 °C and 24 °C (Fig. 5D). The lower peak temperatures in *V. litorea* would suggest that both Q_A_^–^ and Q_B_^–^ are less stable at room temperature in *V. litorea* than in spinach. However, the multiple pathways of recombination (Rappaport and Lavergne, 2009) obviously allow the luminescence-producing minor pathway to suggest destabilization of Q_A_^–^ in *V. litorea* (Fig. 5D) even if the total recombination reaction is slower in *V. litorea* than in spinach (Fig. 5B, C, Supplementary Table S2). The thermoluminescence signal intensity was lower in *V. litorea* than in spinach, suggesting that the luminescence-producing reaction has a low yield in *V. litorea*. The narrow energy gap between Q_A_ and Q_B_ in *V. litorea* favors the probability of an electron residing with Q_A_. Furthermore, a small Q_A_–Q_B_ energy gap also increases the probability that S_3_Q_B_^–^ or S_2_Q_B_^–^ recombine directly and non-radiatively without producing triplet P_680_ and subsequently ^1^O_2_ (Ivanov *et al.*, 2003; Sane *et al.*, 2003, 2012; Ivanov *et al.*, 2008).

We also estimated the amounts of different carotenoids and α-tocopherol in the samples, as these are the most important agents of ^1^O_2_ detoxification in thylakoids (Khorobrykh *et al.*, 2020). The absorption spectra of the pigments from spinach and *V. litorea* thylakoids in DMF suggest that *V. litorea* thylakoids contain more carotenoids, relative to Chl *a*, than spinach, as evidenced by the high absorption in the 460–520 nm region (Fig. 5E). The specific pigments causing the increased absorption in this region in *V. litorea* could not be identified, but possible candidates are the xanthophyll cycle pigment diadinoxanthin (and, to a lesser extent in dark-acclimated samples, diatoxanthin), β-carotene, vaucheriaxanthin, and heteroxanthin (Whittle and Casselton, 1975; Cruz *et al.*, 2015; Streckaite *et al.*, 2018). To get a better idea of the specific compounds related to ^1^O_2_ detoxification, we performed an HPLC analysis of spinach and *V. litorea* thylakoids after methanol extraction. Unfortunately, the lack of standards for pigments such as vaucheriaxanthin, diadinoxanthin, and heteroxanthin did not allow us to confidently identify many compounds that were detected in the HPLC chromatograms (Fig. 5F, Supplementary Fig. S4). Nevertheless, certain differences were noticeable, such as the lack of α-tocopherol in spinach thylakoids as opposed to *V. litorea*, in which it was abundant (Fig. 5F, Table 3). Relative to Chl *a*, *V. litorea* also contained more antheraxanthin (not detected in spinach thylakoids) and β-carotene, but less lutein, violaxanthin, and neoxanthin (not detected in *V. litorea* thylakoids) (Table 3).

**Table 3.** Pigment concentrations of isolated spinach and *V. litorea* thylakoids, as quantified by HPLC after methanol extraction

Pigment	Spinach (µg ml^–1^)	Spinach pigment/Chl *a*	*V. litorea* (µg ml^–1^)	*V. litorea* pigment/Chl *a*
Neoxanthin	84.42±2.84	0.04	n.d.	n.a.
Violaxanthin	1007.7±26.4	0.51	118.91±4.75	0.10
Antheraxanthin	n.d.	n.a.	78.97±3.8	0.07
Lutein	592.97±3.61	0.30	43.6±1.18	0.04
α-tocopherol	n.d.	n.a.	36.29±1.08	0.03
Chl *a*	1979.58±57.31	1	1162.53±43.22	1
Chl *b*	463.89±26.97	0.23	n.d.	n.a.
β-carotene	49.41±4.63	0.02	35.69±1.32	0.03

The data are the means ±SD from three HPLC runs from pooled thylakoids isolated from three different plants/algae flasks. n.d., not detected; n.a., not applicable.

### *In vitro* high-light treatment lowers electron donation to methyl viologen and maximal oxidation of P_700_ in *V. litorea*

**Fig. 6.**
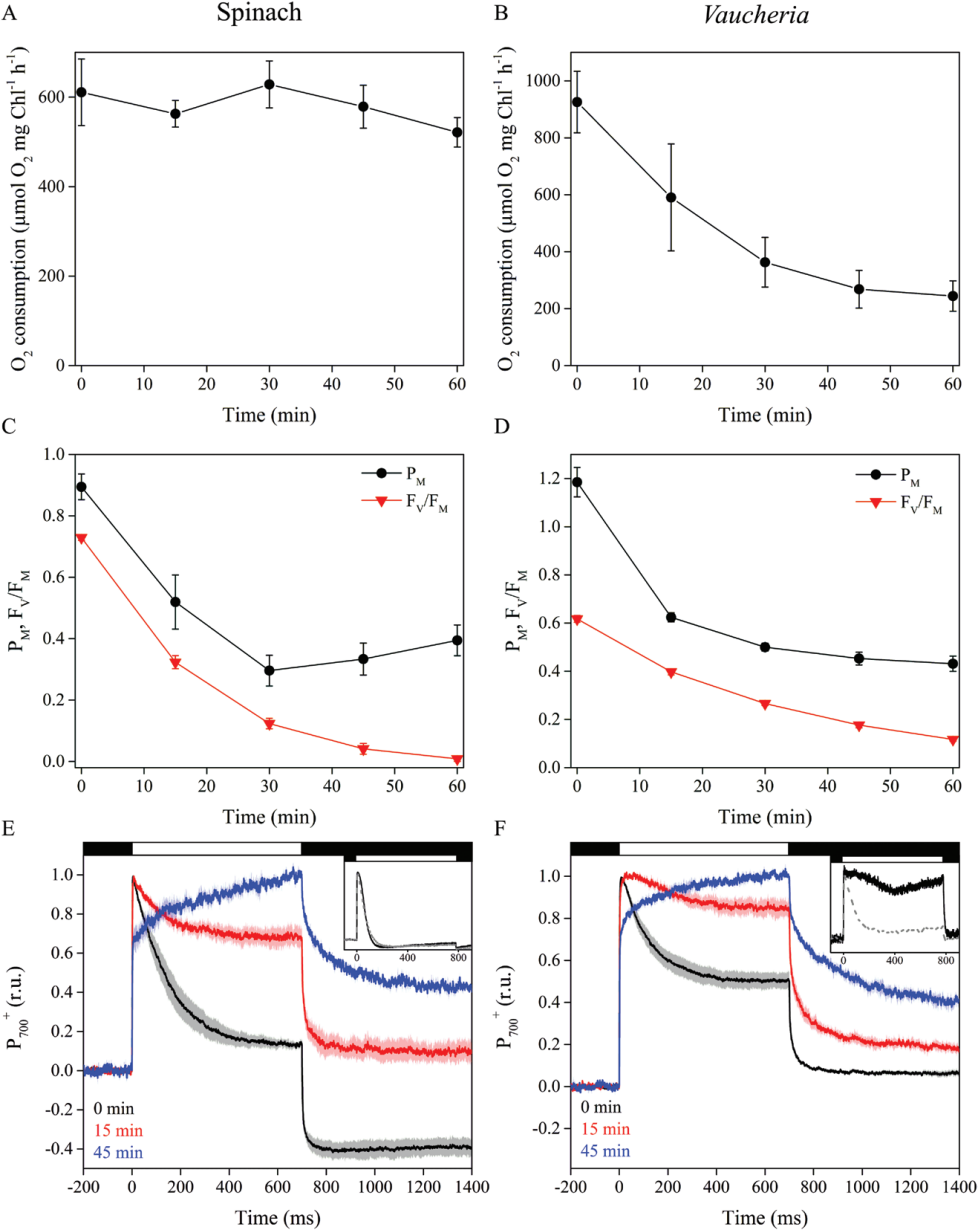
Photoinhibition of PSI in isolated thylakoids of spinach and *V. litorea* during high-light treatment, estimated with oxygen measurements or absorption-based methods. (A, B) Photoinhibition of PSI in (A) spinach and (B) *V. litorea* in the same experimental setup as in Fig. 5. (C, D) Decrease in maximal oxidation of P_700_ reaction center Chl (P_M_) and PSII photochemistry (F_V_/F_M_) during high-light treatment (PPFD 1000 µmol m^–2^s^–1^) in isolated (C) spinach and (D) *V. litorea* thylakoids. (E, F) P_700_ redox kinetics of (E) spinach and (F) *V. litorea* thylakoids during the high-light pulses used for P_M_ determination after 0, 15, and 45 min in photoinhibition treatment. Black and white bars at the top indicate darkness and illumination by the high-light pulse, respectively. The insets show P_700_ redox kinetics from intact spinach leaves and *V. litorea* cells in aerobic (solid black line) and anaerobic (dashed grey line) conditions. Dark control experiments are shown in Supplementary Fig. S4. The P_700_ kinetics in (E, F) have been normalized to stress the form of the curve. All data are means ±SE from at least three biological replicates.

When PSI activity was estimated as electron transfer from 2,6-dichlorophenolindophenol (DCPIP) to methyl viologen (oxygen consumption), spinach PSI remained undamaged during *in vitro* high-light treatment, while *V. litorea* seemed highly susceptible to photoinhibition of PSI (Fig. 6A, B). We repeated the photoinhibition experiment, but this time PSII and PSI activities were monitored by changes in Chl fluorescence and P_700_ absorption. Again, the thylakoid membranes of spinach were more sensitive to photoinhibition of PSII during the high-light treatment than *V. litorea* (Fig. 6C, D). However, this time the PSI functionality of both species decreased similarly when estimated as the maximum oxidation of P_700_ (P_M_). The decrease in P_M_ was strong during the first 15 min (for *V. litorea*) or 30 min (for spinach) of the light treatment, after which P_M_ remained at a somewhat stationary level (Fig. 6C, D). The decrease in P_M_ depended on electron transfer from PSII, as P_M_ did not decrease in high light in spinach thylakoids in the presence of DCMU (Supplementary Fig. S6).

In both spinach and *V. litorea*, redox kinetics of P_700_, measured in thylakoids in aerobic conditions (Fig. 6E, F) were similar to their respective *in vivo* kinetics (Fig. 6E, F, insets), that is, P_700_ in *V. litorea* remained more oxidized during a light pulse than in spinach. Isolating thylakoids from *V. litorea* did, however, cause a decrease in P_700_ oxidation capacity. Unlike in spinach, P_700_ remains oxidized during a high-light pulse in intact *V. litorea* cells if oxygen is present, indicating that alternative electron sinks, such as flavodiiron proteins, function as efficient PSI electron acceptors in *V. litorea* (Fig. 6E, F, insets), probably protecting PSI against the formation of ROS, mainly superoxide or hydrogen peroxide and possibly hydroxyl radical (Allahverdiyeva *et al.*, 2015; Ilík *et al.*, 2017; Shimakawa *et al.*, 2019). In both species, P_700_ redox kinetics changed in the same way during the course of the high-light treatment of isolated thylakoids. The tendency of both species to maintain P_700_ oxidized throughout the high-light pulse in measurements done after 15 min treatment in high light is possibly due to decreasing electron donation caused by photoinhibition of PSII. At the 45 min time point, the damage to PSI was more severe, as indicated by a clear slowing down of P_700_ oxidation that could be associated with problems in electron donation to downstream electron acceptors of PSI such as ferredoxin (Fig. 6E, F).

## Discussion

### Expression of FtsH is at the center of *V. litorea* plastid longevity

Previous studies have shown that the kleptoplasts stemming from *V. litorea* carry out *de novo* protein translation and are generally quite robust inside *E. chlorotica* (Green *et al.*, 2000, 2005; Rumpho *et al.*, 2001). Our transcriptomic analysis of *V. litorea* plastids demonstrates active and regulated transcription of the plastome throughout the 7 days of isolation we tested (Fig. 2), deepening our knowledge about the factors underpinning their native robustness. The amounts of *ftsH* and *psbB* showed very different regulatory patterns, strongly suggesting that these genes do not constitute an operon although they are located after each other and share the same orientation (Fig. 2A).

Our results highlight the increase in the relative amounts of *ftsH* and *tufA* transcripts during a period of several days after the isolation of *V. litorea* plastids. Active transcription of these genes also occurs in the plastids of *E. timida* after a month of starvation (de Vries *et al.*, 2013). FtsH protease is critical for the PSII repair cycle, where it is responsible for degradation of the D1 protein after pulling it out of the PSII reaction center. Recent findings in cyanobacteria, green algae, and higher plants imply that FtsH is also important for quality control of a multitude of thylakoid membrane proteins and thylakoid membrane biogenesis (reviewed by Kato and Sakamoto, 2018). These findings may suggest that the removal of the D1 protein from damaged PSII itself serves to protect from further photodamage and the production of ROS. Even though we were not able to test the genetic autonomy of *V. litorea* plastids inside *E. chlorotica*, the results of our photoinhibition experiments with the long-term-retention slug *E. timida* may serve as a model of photoinhibition in other slug species, as they indicate that the kleptoplasts of *E. timida*, which are stolen from the green alga *A. acetabulum*, possess a genetic toolkit capable of maintaining a PSII repair cycle (Fig. 2C).

We showed that the capacity of *V. litorea* plastids to recover from photoinhibition of PSII in the presence of CHI is nearly unaffected (Fig. 3B). While our CHI experiments on spinach need further exploration in terms of the effects of CHI, studies on the green alga *Chlamydomonas reinhardtii* (which also lacks *ftsH* in its plastome) have shown severe defects in PSII repair both during high light and during subsequent recovery when exposed to CHI (Fig. 3A, Wang *et al.*, 2017). Mutant lines of *C. reinhardtii* have also been used to show that abundant FtsH offers protection from photoinhibition of PSII and enhances the recovery process (Wang *et al.*, 2017). In *C. reinhardtii*, the FtsH hetero-oligomers responsible for D1 degradation are composed of FtsH1 (A-type) and FtsH2 (B-type) (Malnoë *et al.*, 2014). We probed the relative levels of FtsH protein of *V. litorea* during the photoinhibition experiment using antibodies raised against *A. thaliana* A-type (FtsH 1 + 5) and B-type FtsH (FtsH 2 + 8) in the absence and presence of CHI (Fig. 3D). At the end of the recovery period, the CHI-treated cells showed elevated levels of FtsH according to both tested antibodies. The increased abundance of FtsH did not enhance the recovery from photoinhibition of PSII in our experimental setup (Fig. 3B), but our results do point to a tendency of both truly isolated (Fig. 2) and functionally isolated (Fig. 3) *V. litorea* plastids to increase the relative expression of FtsH.

### Low ^1^O_2_ yield does not prevent photoinhibition of PSII but can help maintain efficient repair processes in *V. litorea*

A green alga that is nearly immune to photoinhibition of PSII, *Chlorella ohadii*, has been isolated from the desert crusts of Israel (Treves *et al.*, 2013, 2016). Its resilience against photoinhibition of PSII has largely been attributed to a very narrow energetic gap between Q_A_ and Q_B_, favoring non-radiative charge recombination pathways within PSII that do not lead to ^1^O_2_ production (Treves *et al.*, 2016). While *V. litorea* does not have as small an energetic gap between Q_A_ and Q_B_ as *C. ohadii* (the temperature difference of the *V. litorea* Q- and B-band thermoluminescence peaks was 10 °C, whereas in *C. ohadii* it is only 2–4 °C), PSII charge recombination reactions of *V. litorea* appear to be very slow compared with those of spinach (Fig. 5B–D). Furthermore, the low ^1^O_2_ yield in *V. litorea* (Fig. 5A) suggests that the charge recombination reactions favor the direct non-radiative pathway. The slow charge recombination reactions in *V. litorea* are likely not the only reason behind the low ^1^O_2_ production, as the amounts of ^1^O_2_ detoxification molecules, especially carotenoids and α-tocopherol, affect the measured amount of ^1^O_2_. The cells of *V. litorea* appear to maintain abundant α-tocopherol even after an overnight period of darkness, whereas in spinach we did not detect any α-tocopherol (Fig. 5F, Table 3). Our pigment analyses may suggest that ^1^O_2_-neutralizing compounds are more abundant in *V. litorea* than in spinach, pointing to a tendency to avoid ROS in *V. litorea*. The individual contributions of pigments such as vaucheriaxanthin and heteroxanthin to ^1^O_2_ detoxification in *V. litorea* require further validation. The low ^1^O_2_ yield in *V. litorea* likely factors into the lower dark-corrected rate constant of PSII photoinhibition (*k*_PI_) in comparison to that of spinach thylakoids (Table 2) (Vass, 2011). All of our experiments, however, show that *V. litorea* does experience quite regular levels of PSII photoinhibition. This could indicate that the most important effect of the low ^1^O_2_ yield is protection of the autonomous maintenance machinery of the plastids, as ^1^O_2_ has been shown to be specifically harmful for the PSII repair cycle (Nishiyama *et al.*, 2006).

### Thylakoids of *V. litorea* are highly vulnerable to ROS in the absence of regular stromal electron sinks

Despite the lower rate constant of PSII photoinhibition (Table 2) and ^1^O_2_ yield (Fig. 5A), *V. litorea* thylakoids exhibited drastic oxidative damage to lipids and proteins under high light (Fig. 4B, C). Isolated thylakoids are stripped of the main electron sink of PSI, the Calvin–Benson–Bassham cycle, and comparing P_700_ redox kinetics of *V. litorea* cells and isolated thylakoids (Fig. 6F) reveals that they are also, at least partially, devoid of a Mehler-like reaction that safely reduces oxygen to water without producing superoxide and hydrogen peroxide (Allahverdiyeva *et al.*, 2013). This suggests that catalysts of oxygen reduction in *V. litorea* are likely soluble and therefore were lost during the isolation procedure. Angiosperm plants such as spinach do not rely on a Mehler-like reaction and are susceptible to photoinhibition of PSI in fluctuating light (Shimakawa *et al.*, 2019). The photoprotection of PSI by the Mehler-like reaction has been assigned to enhanced electron sink capacity that lowers the probability of one-electron reduction of oxygen to superoxide by PSI. In comparison to spinach, this would make intact plastids of *V. litorea* less reliant on other ROS-detoxification components that detoxify superoxide and hydrogen peroxide in the water–water cycle (Asada, 1999). Conversely, loss of the Mehler-like reaction during thylakoid isolation would leave the thylakoids highly conducive for ROS production by PSI and very susceptible to oxidative damage of the entire photosynthetic machinery by superoxide, hydrogen peroxide, or hydroxyl radicals. This is likely behind the finding that *V. litorea* thylakoids lose the ability to reduce methyl viologen in a high-light treatment that does not affect spinach thylakoids (Fig. 6A, B). When damage to PSI was estimated as a decrease in P_M_, spinach and *V. litorea* thylakoids showed very similar responses to high light, with both species exhibiting a decrease in PSI activity until electron donation from PSII was diminished due to photoinhibition of PSII (Fig. 6C, D), as suggested previously (Sonoike, 1995, 1996). This, in addition to the highly similar changes in the redox kinetics of P_700_ during the photoinhibition treatment (Fig. 6E, F) between the two species, would suggest that the decrease in oxygen consumption in *V. litorea* thylakoids is caused by further, more severe, damage to PSI than the process causing the decrease in P_M_. The nature of this reaction is not known but it may be caused by the production of ROS due to continuing electron flow through PSI in thylakoids of *V. litorea* exhibiting a low rate constant of PSII photoinhibition (Table 2) and normally relying on stromal electron acceptors for the protection of PSI.

PSI of *V. litorea* is not particularly prone to photoinhibition, but our results do confirm that the electron sinks of photosynthesis must be functional to avoid large-scale oxidative damage. This is especially relevant for animals that host a foreign organelle where uncontrolled ROS production is detrimental (de Vries *et al.*, 2015). Our recent results in the long-term-retention sea slug *E. timida* show that oxygen functions as an alternative electron sink in the slug’s plastids (Havurinne and Tyystjärvi, 2020), but whether the record-holding species *E. chlorotica* utilizes the oxygen-dependent electron sinks provided by *V. litorea* (Fig. 6F, inset) remains to be tested. As for the main electron sink of photosynthesis, the carbon fixation rates of the plastids inside *E. chlorotica* are comparable to the rates measured from *V. litorea* cells after incorporation (Rumpho *et al.*, 2001), suggesting that carbon fixation is not a problem in *E. chlorotica*.

### Conclusions

The plastids of *V. litorea* are genetically more autonomous than those of embryophytes, containing genes that help to maintain plastid functionality. After isolation of the plastids, the relative expression of the translation elongation factor EF-Tu and the central maintenance protease FtsH increases—a phenomenon that may be important for plastid longevity in the foreign cytosol of a sea slug. Low ^1^O_2_ yield protects the functionality of the plastid-encoded maintenance machinery and may slow down photoinhibition of PSII. Interruption of oxygen-dependent alternative electron sinks upstream of PSI leads to large-scale oxidative damage in *V. litorea*, suggesting that carbon fixation, the main electron sink of photosynthesis, needs to remain in near-perfect working order to avoid destruction of the plastids. Our results support decades-old data (Trench *et al.*, 1973a, b) suggesting that the native stability and associated peculiar functionality of the plastids themselves hold the key to long-term kleptoplast longevity in sacoglossans. Nature has evolved an elaborate suite of photoprotective mechanisms, and the unique animal–kleptoplast association allows us to explore them and even identify new ones.

## Supplementary data

The following supplementary data are available at *JXB* online.

Table S1. List of primers used in the qPCR experiment.

Table S2. Modeled S-state distribution of the OEC in spinach and *V. litorea.*

Fig. S1. Relative amounts of *rbcL* and *psaA* transcripts during plastid isolation.

Fig. S2. EPR spectra from spinach and *V. litorea* thylakoids.

Fig. S3. Dark control treatments of *in vitro* PSII photoinhibition in spinach and *V. litorea.*

Fig. S4. HPLC chromatograms from spinach and *V. litorea* thylakoids,

Fig. S5. Dark control treatments of *in vitro* PSI and PSII photoinhibition in spinach and *V. litorea.*

Fig. S6. *In vitro* PSI and PSII photoinhibition in DCMU-treated spinach thylakoids.

erab216_suppl_Supplementary_File001

## Data Availability

The data that support the findings of this study are openly available in Mendeley Data at http://doi.org/10.17632/535dcxjt2d.1; Havurinne *et al.* (2021).

## Abbreviations

^1^O_2_: singlet oxygen
CHI: cycloheximide
DCBQ: 2,6-dichloro-1,4-benzoquinone
DCMU: 3-(3,4-dichlorophenyl)-1,1-dimethylurea
DCPIP: 2,6-dichlorophenolindophenol
DMF: *N*,*N*-dimethylformamide
EPR: electron paramagnetic resonance
F_V_/F_M_: maximum quantum yield of PSII photochemistry
*k*_PI_: rate constant of PSII photoinhibition
MDA: malondialdehyde
OEC: oxygen-evolving complex of PSII
P_680_: reaction center Chl of PSII
P_700_: reaction center Chl of PSI
P_M_: maximum oxidation of P700
PPFD: photosynthetic photon flux density
PSI: photosystem I
PSII: photosystem II
qPCR: quantitative real-time PCR
ROS: reactive oxygen species
TEM: transmission electron microscope
Tyr_D_^+^: oxidized tyrosine-D residue of PSII
