# Supplementary material for: Genetic autonomy and low singlet oxygen yield support kleptoplast functionality in photosynthetic sea slugs
Source: J Exp Bot. 2021 May 15;72(15):5553–68. doi: 10.1093/jxb/erab216 (PMC8318255; doi:10.1093/jxb/erab216)
Supplement: erab216_suppl_Supplementary_File001 [file erab216_suppl_supplementary_file001.pdf]

Supplementary data

Supplementary table S1. The list of primers designed for quantitative real-time PCR analysis of transcription in isolated *V. litorea* plastids.

Symbol	Description	Primer (5’→3’)
ftsH	FtsH protease subunit	for- TGATGTTGTTTTTGATGATGTTGC<br>rev- ACTCCTTTTGGTATTTTAGCACCT
psaA	PSI protein PsaA	for-TGGACTGCTATTGGTGGTTT<br>rev-CCATTCAAGTTTAGGTGCTGCT
psbA	PSII protein D1	for-ATTCCCACTCACGACCCATA<br>rev- AAACAACATCATTTCTGGTGCT
psbB	PSII protein CP47	for-ATGGGCTGGTTCAATGGCTT<br>rev-GCTACACCCTCAAAACTCCA
psbC	PSII protein CP43	for-TGGTCTGGAAATGCTCGTCTT<br>rev-CAACGCCCCATCCTAAAGTA
psbD	PSII protein D2	for-TGGACAAAATCAAGAACGAGGT<br>rev-ACCAACCAATAAATACGAAGCGA
psbH	PSII protein PsbH	for-AAAAGTTGCTCCTGGTTGGG<br>rev-ATATTTTGCCAATCAACATCTACA
rbcL	RuBisCo large subunit	for-CGCTCTCTCCAACGCATAA<br>rev-GGACTTCGTGGTGGTTTAGATTT
tufA	Translation elongation factor EF-Tu	for-TATCTACCCATTCATTATCCCCTTT<br>rev-ATTCCTATTTGCCCAGGTTCAG

Supplementary table S2. Percentage distribution of the S-states of the OEC in isolated thylakoids from spinach and *V. litorea* after different preflash treatments prior to measuring flash induced oxygen evolution. The flash oxygen data in Figure 5B was modeled essentially as described in Antal *et al*., (2009) to estimate the S-state distribution.

	S_0 % (±SE)	S_1 % (±SE)	S_2 % (±SE)	S_3 % (±SE)
Spinach 10 min dark	0.01 (±0.01)	71.01 (±2.78)	24.54 (±1.95)	4.44 % (±1.15)
Spinach preflash + 10 s dark	0	32.30 (±9.36)	57.28 (±5.83)	10.42 (±3.63)
Spinach preflash + 100 s dark	0	65.30 (±0.17)	26.32 (±0.07)	8.39 (±0.24)
Vaucheria 10 min dark	6.84 (±3.20)	63.44 (±3.66)	24.65 (±3.70)	5.08 (±3.20)
Vaucheria preflash + 10 s dark	0.02 (±0.01)	24.69 (±1.19)	60.62 (±0.59)	14.66(±0.83)
Vaucheria preflash + 100 s dark	0.01 (±0.01)	45.21 (±2.03)	39.18 (±0.92)	15.61 (±2.53)

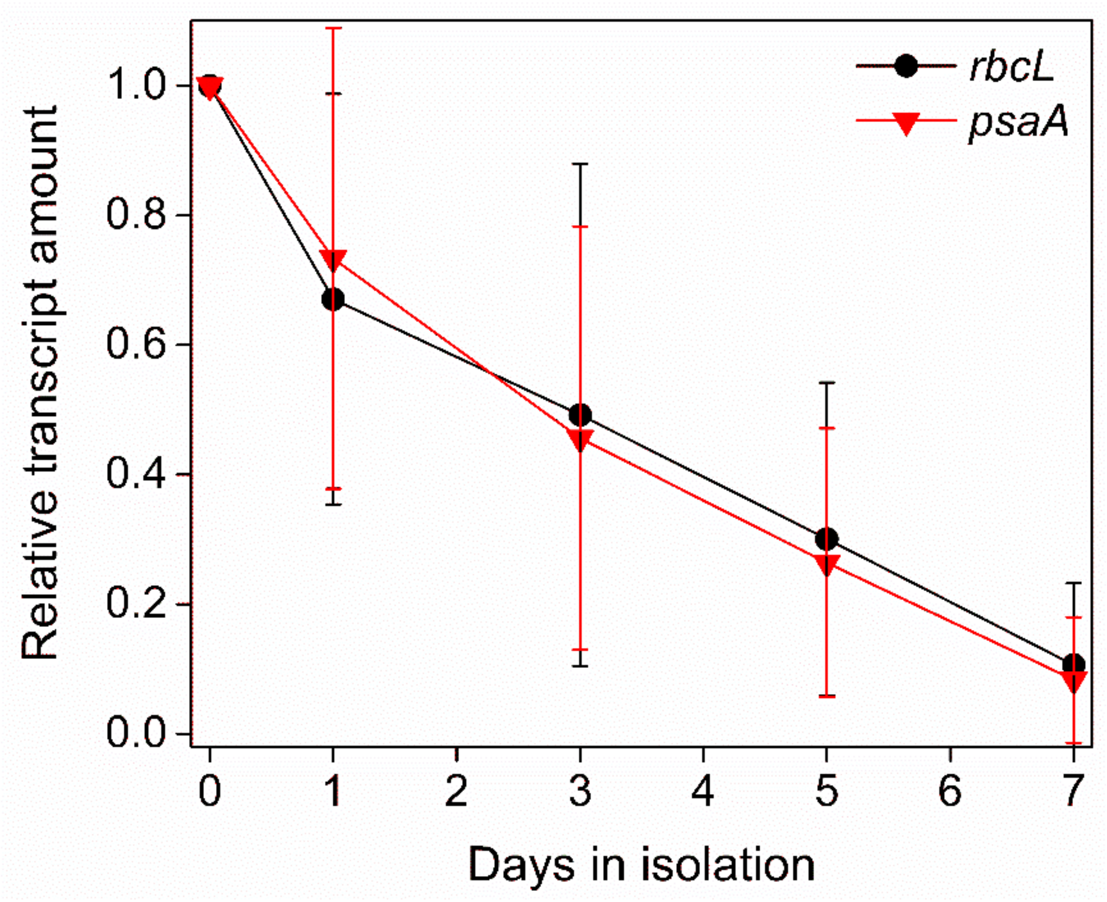

Supplementary Figure S1. Loss of *rbcL* and *psaA* transcripts during incubation of the isolated plastids. Relative transcript amounts were estimated from the cycle threshold values of the two genes as $2^{(CT0 - CTt)}$, where CT0 and CTt stand for cycle threshold at timepoint 0 and at different timepoints of the isolation period, respectively. Each data point represents an average from three biological replicates and the error bars show standard deviation.

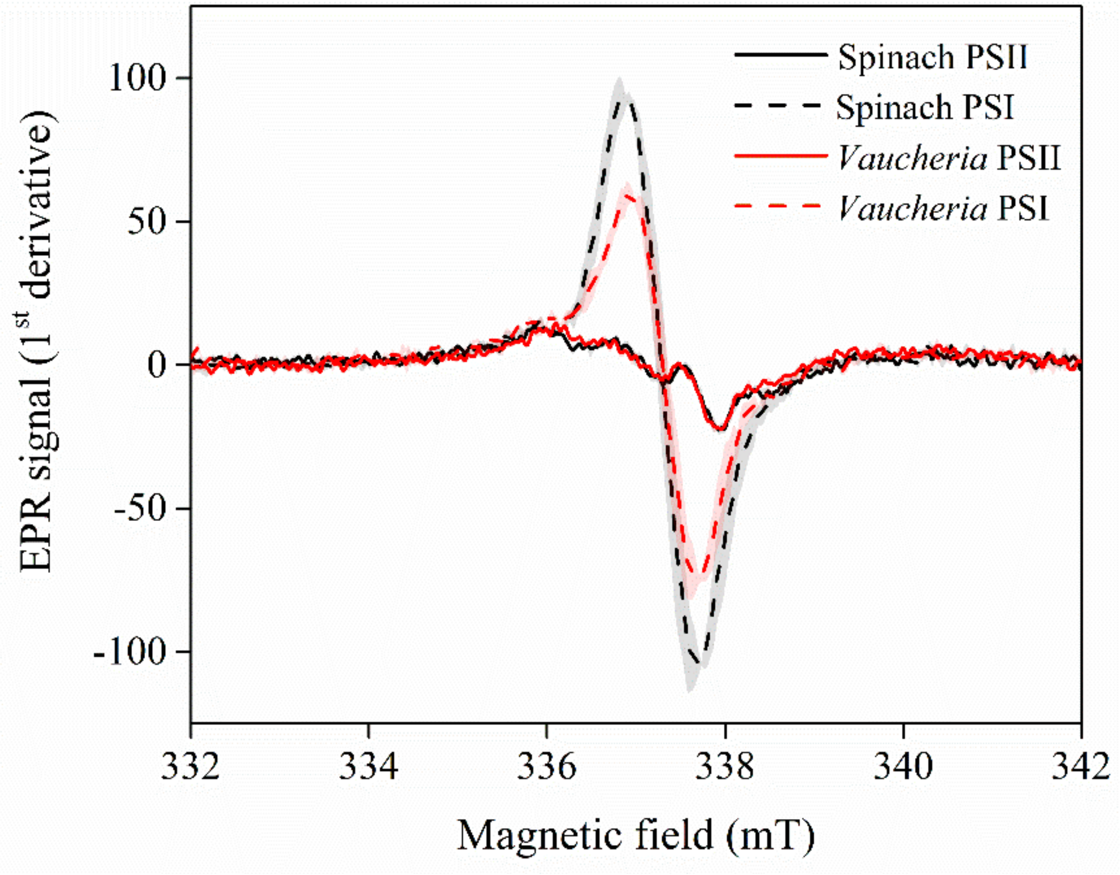

Supplementary Figure S2. EPR spectra of PSII (Tyr_D^+) and PSI (P_{700}^+) in spinach and *V. litorea* thylakoids. All spectra were measured from isolated thylakoid samples containing 2000 µg total Chl ml^{-1}. Each curve represents an average of three independent biological replicates and the shaded areas around the curves represent SE.

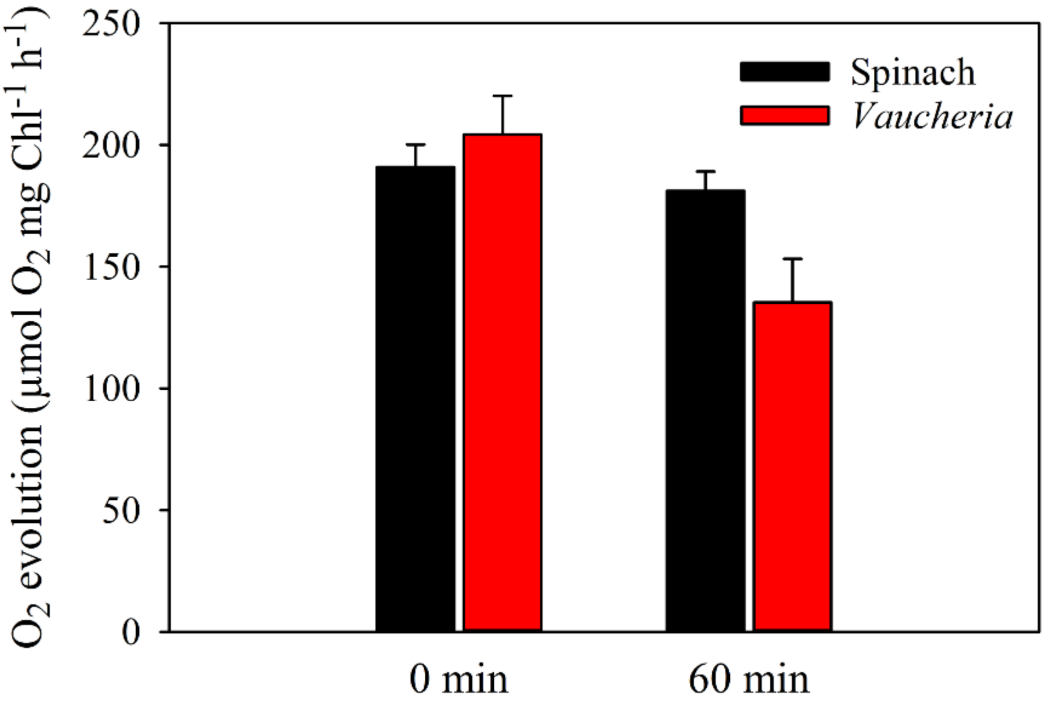

Supplementary Figure S3. Dark control treatments of the *in vitro* photoinhibition experiments shown in Fig. 4A of the main text. PSII activities of spinach (black) and *V. litorea* (red) at the onset and after a 60 min dark treatment at 22 °C in photoinhibition buffer. Oxygen evolution was measured in the presence of 0.5 mM DCBQ and hexacyanoferrate(III) from samples containing 20 µg total Chl ml^{-1}. Rate constant of PSII dark inactivation was 0.001 min^{-1} for spinach and 0.007 min^{-1} for *V. litorea*. Each data point represents an average of three biological replicates and the error bars indicate SE.

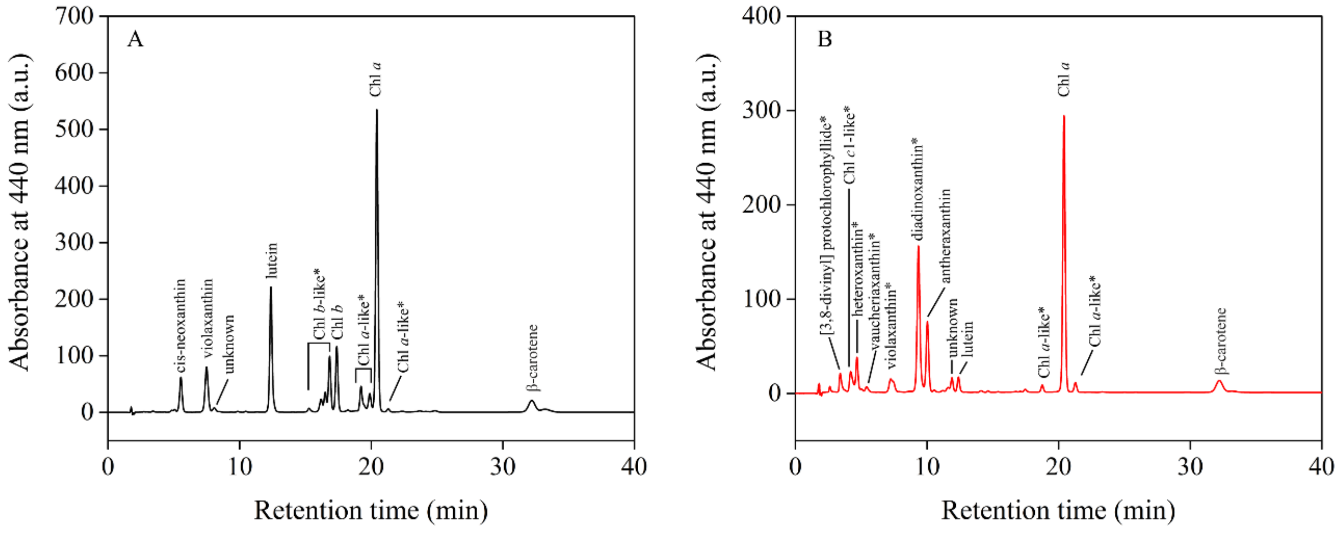

Supplementary figure S4. HPLC chromatograms from spinach (A) and *V. litorea* (B) thylakoids after methanol extraction. The asterisks mark pigments that were identified based on literature, but were not quantified in the present work. The chromatograms represent triplicate HPLC runs from pooled thylakoids isolated from three different plants/algae flasks.

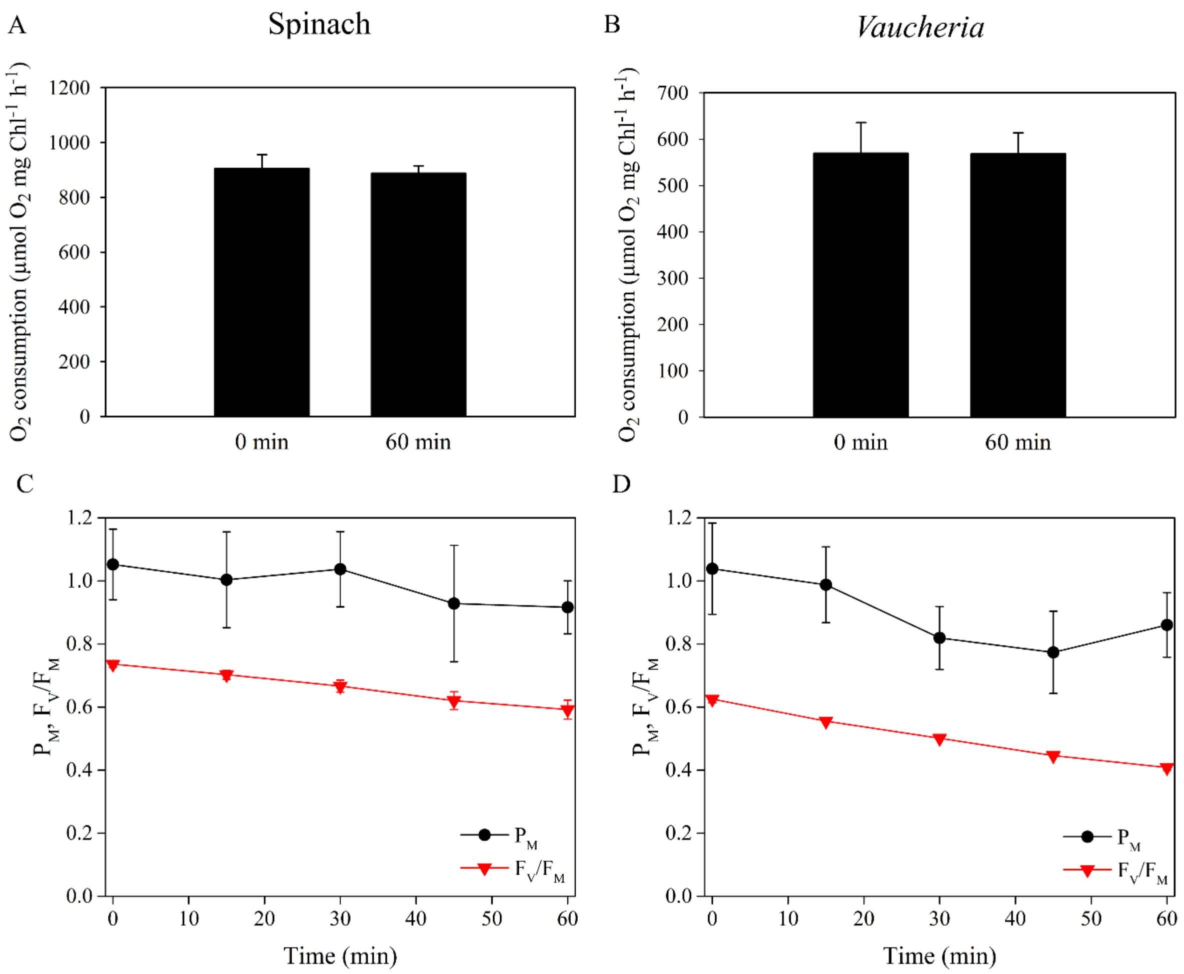

Supplementary figure S5. Dark control treatments of the *in vitro* photoinhibition experiments shown in Fig. 6 of the main text. (A, B) PSI activities of spinach and *V. litorea* during a 60 min dark incubation period in photoinhibition buffer, measured as oxygen consumption. (C, D) PSI and PSII activities in isolated spinach and *V. litorea* thylakoids during a 60 min dark treatment, as estimated by maximal oxidation of P_{700} (P_M; black) and F_V/F_M (red), respectively. All data are averages from a minimum of three biological replicates and error bars indicate SE.

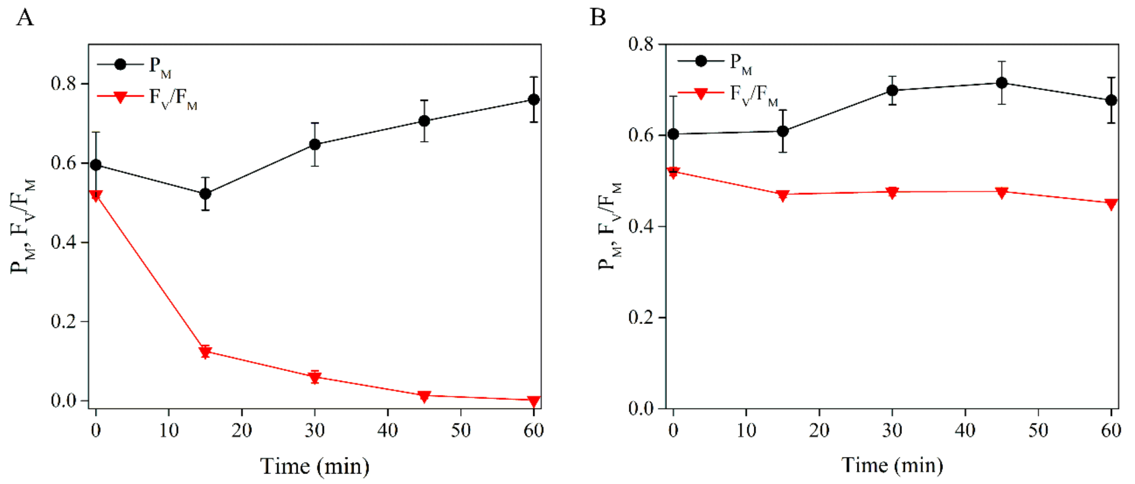

Supplementary figure S6. DCMU prevents photoinhibition of PSI in isolated spinach thylakoids. (A) Maximal oxidation of P_{700} (P_M; black) and maximum quantum yield of PSII (F_V/F_M; red) were measured during a 60 min high-light treatment (PPFD 1000 µmol $m^{-2}s^{-1}$) in DCMU treated spinach thylakoids. (B) Dark control experiments using the same setup as in (A). All data are averages from four biological replicates and the error bars indicate SE.
